# Mr. Swan on the Sympathetic Nerve

**Published:** 1847-04

**Authors:** 


ART. IV.—*The Nature and Faculties of the Sympathetic Nerve.* By JOSEPH SWAN.—*London,* 1847. 8vo, pp. 55.

WE have every respect for Mr. Swan as an able and industrious anatomist; but we cannot feel a similar confidence in his physiological deductions. There is a vagueness about his language, when he is speaking of *function,* which forms a striking contrast to the clearness of his descriptions of *structure,* and which frequently (as it seems to us) misleads not only his readers but himself. In the preface to the pamphlet before us, he very clearly points out the difficulties attendant on the study of this division of the nervous system, and indicates the sources whence information may be obtained regarding its offices in the system. And in the second chapter, in which he describes the chief variations in the structure and arrangements of the sympathetic system in different classes of vertebrata, he gives many interesting anatomical particulars, which are valuable as data for physiological reasoning. But at the very commencement of his inquiries into the operations of the sympathetic, he betrays the want of those clear ideas of the relation between the animal and organic functions, on which alone any correct notion of its influence on the latter can be erected. "The great object of the sympathetic nerve," he says (p. 4), "is to furnish the parts it supplies with an appropriate nervous excitement of such a quality as will ensure their functions without disturbing any other portion of the nervous system." Now the whole gist of the matter lies in the simple phrase "ensure their functions." Every one knows that the sympathetic system is distributed chiefly to the heart and sanguiferous system, and to the intestinal canal. Does Mr. Swan assert that the heart cannot beat,—that the arteries, capillaries, and veins cannot convey blood,—that the several parts which they supply cannot draw from the blood the materials of their growth and nutrition,—and that the peristaltic movements of the intestinal tube cannot continue,—without the constant influence of this system of nerves? Such would appear to be his meaning, from various expressions scattered through the pamphlet, though he nowhere (that we have discovered) formally states these views. The following passage, which succeeds the one we have just quoted, will give a fair specimen of Mr. Swan's style; and we think that our readers' judgment upon it will correspond with our own:

"It connects in different degrees all the parts of the nervous system as an harmonious whole, but brings them in so slight a degree in communion with the sensory, as to allow only a perceptibility that can appreciate and respond to impulses without permitting them to proceed beyond the viscera. By preventing sensation, it becomes favorable to the production of involuntary motion, so that impulses on the lining membrane of the viscera, when sufficiently strong, are responded to, and the contraction of the muscular coat takes place. For these purposes it has a peculiar conformation which differs more or less from [that of] the other parts of the nervous system. Although it communicates with several cerebral nerves, it assimilates most to the fifth and spinal nerves. From these and their centres it probably derives some of their essential or diffusive influence for fortifying its vital powers, but admits only just as much of their faculties as corresponds with the functions of the parts it supplies. It is not less extensive in any of the four superior classes of animals, in proportion to the parts it actuates, but its structure is more or less complex, and in the same degree its faculties are more distant from, or approach nearer to, those of the rest of the nervous system, and accordingly are more or less independent. When its faculties are insufficient for the organs, having more complicated functions than those it generally promotes, branches of the fifth, the par vagum, and spinal nerves, are combined with portions of it; or when any organs, supplied by cerebral and spinal nerves, require a more general and higher excitement from the heart and arteries, they receive more branches from the sympathetic." (pp. 4, 5.)

We might easily show the vague and unsubstantial character of every one of the statements contained in this paragraph. What clear notion can be attached to "a perceptibility that can *appreciate* and respond to impulses," if no consciousness of those impulses be excited? What proof exists, that any such reflex action is performed by the ganglia of the sympathetic system? What definite meaning can be drawn from the "essential or diffusive influence for fortifying its vital powers," supposed to be derived by the sympathetic from the fifth cerebral and the spinal nerves? What ground is there for the assumption that the "faculties" of the sympathetic are "insufficient for the organs" it supplies, save the fact that other nerves are transmitted to them?—or what indication exists of the "more general and higher excitement from the heart and arteries" asserted by Mr. Swan to be required by some organs, save the larger number of branches of the sympathetic proceeding to them? A clearer case of reasoning in a circle never came under our notice. We might quote an abundance of passages of the same kind; and upon the whole we feel constrained to say, that, except the few anatomical details contained in the second chapter, the pamphlet contains nothing but a series of vague and unmeaning speculations, couched in language which is a great deal too positive.

